# Breast tumour localisation using Iodine seeds in the UK: the first 100 patients

**DOI:** 10.1186/bcr3760

**Published:** 2015-11-05

**Authors:** Nidhi Sibal, Nerys Forester

**Affiliations:** 1 Newcastle Teaching Hospitals, Newcastle, UK

## Introduction

Wire localization techniques for impalpable breast tumours require wire placement ideally on the day of surgery. Tumour localization using iodine-125 seeds allows tumour localization to occur prior to surgery, improving both work flow dynamics and the patient experience. Newcastle Hospitals Trust is the first centre in the UK to adopt this technique. Here we present our initial experience of the first 100 patients to undergo wire-free surgery.

## Methods

From September 2014, data were prospectively collected on all patients undergoing iodine seed tumour localization. Seeds were placed under ultrasound guidance into tumours identifiable on ultrasound between 7 and 14 days preoperatively. Seeds were removed with the tumour after intraoperative localization using a gamma probe.

## Results

Our first 100 patients are included in this initial analysis. The majority of patients had a wide local excision, with 10 undergoing therapeutic mastectomy. Thirteen patients returned to theatre for positive margins or completion mastectomy, depending on the final pathology. No seeds were lost during use. One patient had a second tumour identified at the time of seed placement which required wire localization. No radiological complications occurred. Introduction of iodine seeds improved radiological workflow, with creation of a planned outpatient 'seed list', remote from the day of surgery and radiological high demand times.

## Conclusion

Iodine seed tumour localization in the UK is achievable, patient friendly and has great benefits for radiologists in terms of department workflow. Noticeably, patients (and surgeons) appear much more relaxed since the introduction of this technique and initial patient satisfaction surveys have been positive.

